# Highly accelerated free-breathing real-time myocardial tagging for exercise cardiovascular magnetic resonance

**DOI:** 10.1186/s12968-023-00961-w

**Published:** 2023-10-02

**Authors:** Manuel A. Morales, Siyeop Yoon, Ahmed Fahmy, Fahime Ghanbari, Shiro Nakamori, Jennifer Rodriguez, Jennifer Yue, Jordan A. Street, Daniel A. Herzka, Warren J. Manning, Reza Nezafat

**Affiliations:** 1 Department of Medicine (Cardiovascular Division), Beth Israel Deaconess Medical Center and Harvard Medical School, 330 Brookline Ave., Boston, MA 02215 USA; 2 Case Western Reserve University, Cleveland, OH USA; 3 Department of Radiology, Beth Israel Deaconess Medical Center and Harvard Medical School, Boston, USA

**Keywords:** Real-time tagging, Highly accelerated, Deep learning

## Abstract

**Background:**

Exercise cardiovascular magnetic resonance (Ex-CMR) myocardial tagging would enable quantification of myocardial deformation after exercise. However, current electrocardiogram (ECG)-segmented sequences are limited for Ex-CMR.

**Methods:**

We developed a highly accelerated balanced steady-state free-precession real-time tagging technique for 3 T. A 12-fold acceleration was achieved using incoherent sixfold random Cartesian sampling, twofold truncated outer phase encoding, and a deep learning resolution enhancement model. The technique was tested in two prospective studies. In a rest study of 27 patients referred for clinical CMR and 19 healthy subjects, a set of ECG-segmented for comparison and two sets of real-time tagging images for repeatability assessment were collected in 2-chamber and short-axis views with spatiotemporal resolution 2.0 × 2.0 mm^2^ and 29 ms. In an Ex-CMR study of 26 patients with known or suspected cardiac disease and 23 healthy subjects, real-time images were collected before and after exercise. Deformation was quantified using measures of short-axis global circumferential strain (GCS). Two experienced CMR readers evaluated the image quality of all real-time data pooled from both studies using a 4-point Likert scale for tagline quality (1-excellent; 2-good; 3-moderate; 4-poor) and artifact level (1-none; 2-minimal; 3-moderate; 4-significant). Statistical evaluation included Pearson correlation coefficient (*r*), intraclass correlation coefficient (ICC), and coefficient of variation (CoV).

**Results:**

In the rest study, deformation was successfully quantified in 90% of cases. There was a good correlation (*r* = 0.71) between ECG-segmented and real-time measures of GCS, and repeatability was good to excellent (ICC = 0.86 [0.71, 0.94]) with a CoV of 4.7%. In the Ex-CMR study, deformation was successfully quantified in 96% of subjects pre-exercise and 84% of subjects post-exercise. Short-axis and 2-chamber tagline quality were 1.6 ± 0.7 and 1.9 ± 0.8 at rest and 1.9 ± 0.7 and 2.5 ± 0.8 after exercise, respectively. Short-axis and 2-chamber artifact level was 1.2 ± 0.5 and 1.4 ± 0.7 at rest and 1.3 ± 0.6 and 1.5 ± 0.8 post-exercise, respectively.

**Conclusion:**

We developed a highly accelerated real-time tagging technique and demonstrated its potential for Ex-CMR quantification of myocardial deformation. Further studies are needed to assess the clinical utility of our technique.

**Supplementary Information:**

The online version contains supplementary material available at 10.1186/s12968-023-00961-w.

## Background

Cardiovascular disease is the leading cause of morbidity and mortality [1]. Cardiac magnetic resonance (CMR) imaging offers valuable imaging biomarkers of myocardial deformation, which can be helpful for prognosis evaluation [2–5]. Myocardial tagging is the noninvasive reference standard for assessing left ventricular (LV) myocardial deformation but has limitations due to tagline contrast degradation [6–8]. Nevertheless, the main advantage of tagging is that myocardial deformation is directly measured by physical and magnetic properties of the tissue. Alternative techniques, such as displacement encoding with stimulated echoes (DENSE), harmonic phase (HARP), and strain encoding (SENC), also face challenges such as low signal-to-noise ratio (SNR) and require dedicated sequences not widely available [9]. Cine imaging is commonly used for LV assessment, and cine measures of myocardial deformation, such as strain, are widely used in the research [2, 4, 5, 10].

Assessing myocardial deformation during cardiovascular stress is an area of growing interest, with pharmacological stimulation being a standard approach [11–16]. However, pharmacological stress does not fully replicate the cardiovascular response to physical stress [17–19] and lacks crucial prognostic data [20, 21]. Exercise combined with imaging (Ex-CMR) presents a promising yet technically challenging alternative [22]. Evaluating LV deformation in Ex-CMR using cine images can be unreliable due to increased heart rates and motion during physiological stress. In contrast, tagging images provide intrinsic, well-defined structural landmarks that may facilitate detailed motion analysis [23]. Moreover, Ex-CMR at 3 T offers increased tag contrast duration resulting from lengthened myocardial T1, which mitigates the limitation of tagline contrast degradation. Early tagging Ex-CMR studies using electrocardiogram (ECG)-segmented sequences aimed to reduce breath-hold time [24–26]. However, temporal resolution was suboptimal (i.e., > 30 ms) [27], and optimal temporal resolution was often achieved at the expense of spatial resolution [28]. Further, a stable ECG signal was needed to segment k-space data during the reconstruction [25, 26]. Therefore, ECG issues during maximal physiological stress could compromise image quality.

Acceleration methods based on k-space undersampling could enable free-breathing real-time tagging without needing k-space segmentation. Parallel imaging is widely used in CMR with acceleration rates less than 3 [29–31]. Compressed sensing (CS) [32] has recently been incorporated into vendor applications and allows tagging with higher acceleration rates than parallel imaging [33]. However, rates beyond four result in artifacts and degradation of tagline quality [33]. Deep learning (DL) techniques are increasingly being utilized to enhance image acceleration, potentially allowing for even higher rates [34–38]. For example, these methods enable the omission of high-frequency regions in k-space without causing a significant loss in the spatial resolution [39]. Despite these advances in image acceleration, current tagging gradient-echo sequences suffer from reduced SNR at high acceleration rates. Alternatively, a tagging sequence based on balanced steady-state free-precession (bSSFP) would have a higher SNR that could be traded for a higher acceleration [40–42].

We sought to develop and implement a highly accelerated bSSFP-based free-breathing real-time tagging sequence to enable post-exercise evaluation of cardiac deformation. Initially, the free-breathing real-time sequence was evaluated at rest and compared with an ECG-gated tagging sequence with scan/re-scan reproducibility assessment. Subsequently, the feasibility of using the free-breathing real-time bSSFP tagging was demonstrated in a pilot Ex-CMR study.

## Methods

ECG-segmented and real-time tagging bSSFP sequences were developed to evaluate myocardial deformation. Sequences were deployed with identical parameters to enable an adequate comparison. Real-time tagging was implemented with 12-fold acceleration by combining CS and DL image reconstruction. Quantification of myocardial deformation based on real-time tagging images was evaluated at rest and after the completion of an Ex-CMR protocol.

### Imaging pulse sequences

**Fig. 1:**
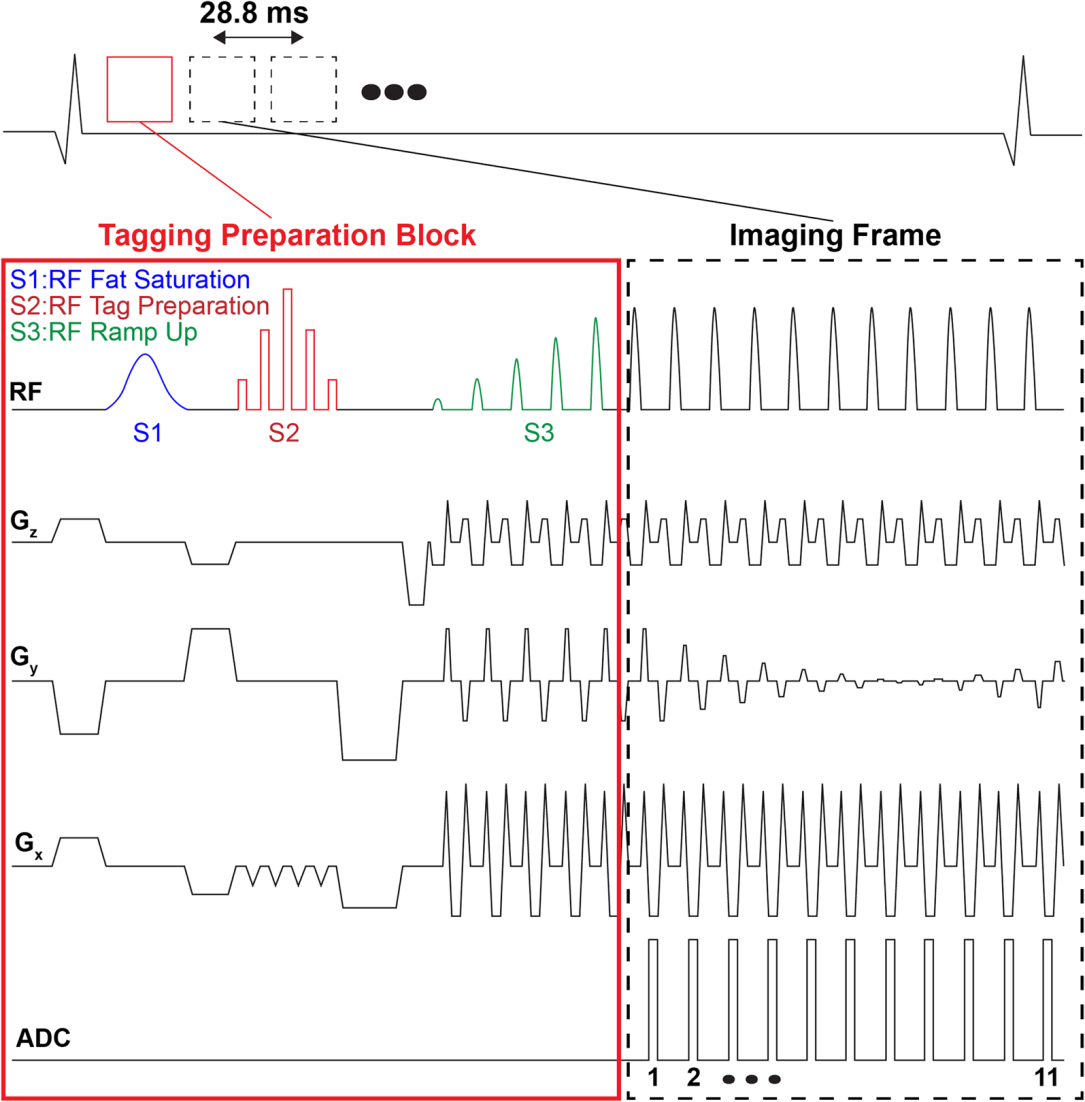
Highly accelerated real-time tagging at 3 T. Steady-state free-precession imaging has a higher signal-to-noise ratio than gradient-echo and is thus more robust to high acceleration rates. Tag lines were perpendicular to phase-encoding to preserve harmonics. Off-resonance artifacts were reduced by using fat-saturation (S1) and steady-state ramp-up (S3) pulses. Only five pulses were used to minimize the delay between tag preparation (S2) and the first imaging frame

We developed the real-time tagging sequence by adding a bSSFP-based highly accelerated Cartesian imaging block after tagging preparation (Fig. 1**)**. In this technique, in addition to acceleration by undersampling, further acceleration was achieved by omitting or truncating the high-frequency k-space regions along the phase encoding (PE) direction. Thus, the tagging gradients were applied perpendicular to the PE direction, preserving spatial harmonics solely along the readout direction. Furthermore, bSSFP ramp-up magnetization catalyzing pulses were added before imaging to reduce off-resonance artifacts during the approach to a steady state. When excess fat around the heart was a concern, a fat saturation pulse was added before tagging preparation to avoid chemical shift artifacts that may interfere with the visualization of the deformed tagging pattern and complicate image analysis.

Fat saturation was carried out upon detection of the cardiac trigger by applying a spectrally selective inversion recovery pulse with width = 5.1 ms and flip angle = 110^°^. Five binomially distributed (1-3-4-3-1) radiofrequency pulses were applied to create a tagging line pattern with 8 mm spacing. Applying five linearly increasing ramp-up pulses before the onset of imaging was sufficient to reduce artifacts while minimizing the time delay after applying the tags, which was important during post-exercise due to elevated heart rates. The total imaging delay after cardiac trigger detection was 38 ms.

The bSSFP-based imaging sequence was implemented with twofold outer k-space truncation along the PE direction and incoherent random sixfold undersampling. This implementation resulted in a combined 12-fold acceleration rate. Two-chamber and short-axis tagging images were collected at the basal, mid-ventricular, and apical levels with the following imaging parameters: matrix size = 176 × 176, reconstructed spatial resolution = 2.0 × 2.0 mm^2^, slice thickness = 8 mm, distance factor = 150%, bandwidth = 1235 Hz/Px, TE = 1.1 ms, TR = 2.62 ms, temporal resolution = 28.8 ms, asymmetric echo and flip angle = 30°. The acquisition window for two-chamber images was 1.2 s, while for each short-axis slice was 2 s to allow magnetization recovery before acquisition of the next slice.

We implemented an additional bSSFP-based breath-hold ECG-segmented sequence for comparison. Imaging was divided over eight heartbeats to enable an implementation whose imaging parameters were identical to those used for real-time tagging. An $\alpha$/2 pulse was added TR/2 after imaging was completed for a given heartbeat to store the steady-state magnetization and minimize ghosting artifacts resulting from the interruption of the steady state by the tagging block. Images were acquired with GeneRalized Autocalibrating Partial Parallel Acquisition (GRAPPA) rate 2, and retrospective ECG gating with 25 calculated cardiac phases.

#### Real-time tagging image reconstruction

**Fig. 2:**
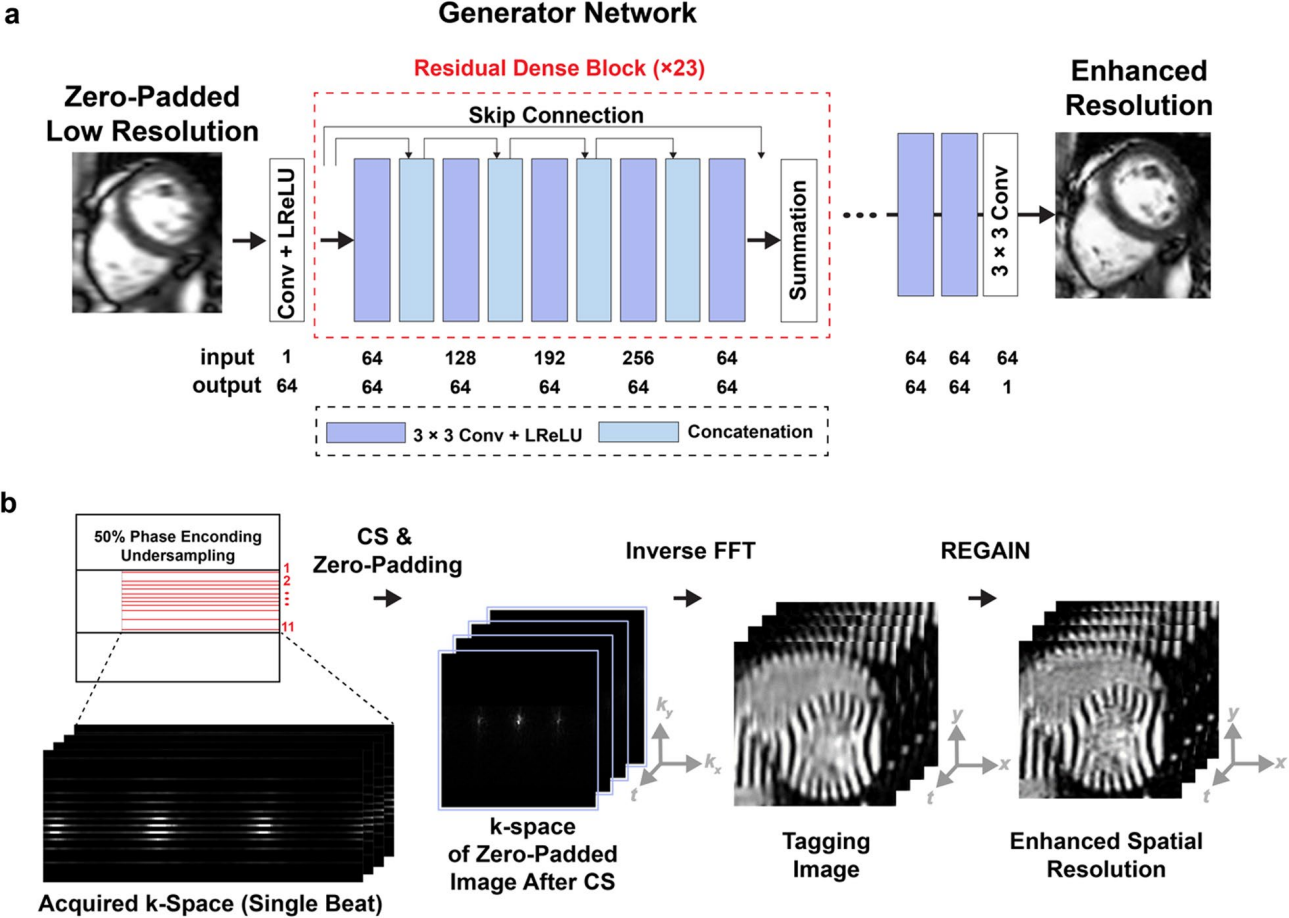
Tagging imaging reconstruction workflow. **a** A resolution enhancement generative adversarial inline network (REGAIN) architecture was previously trained to deblur cine images. REGAIN was developed using breath-hold electrocardiogram-segmented segmented cine for training. **b** Reconstruction consisted of vendor-provided compressed sensing (CS) reconstruction per beat to create images from sixfold under-sampled k-space data. Resulting blurred images (with twofold reduced phase encoding spatial resolution) were used as input to REGAIN to deblur low-resolution CS-reconstructed images. The REGAIN recon was used to reconstruct tagging data without any modification. 12-fold accelerated k-space data were reconstructed in-scanner in real-time, and images were available upon completion of scan

Image reconstruction was performed using a resolution enhancement generative adversarial inline network (REGAIN), a DL-based approach [43]. First, tagging images were reconstructed with vendor-provided CS and zero-padding along the PE direction to create an image with full resolution, albeit with significant blurring. Subsequently, images with spatial resolution analogous to collecting a full-resolution image were generated by applying REGAIN (Fig. 2). Briefly, REGAIN enhances the spatial resolution of images from the low-resolution inputs, which enables further acceleration based on the omission of PE lines. The network was trained using a pair of ground truth and synthesized zero-padded low-resolution cine images generated using raw k-space data (Fig. 2a).

In this study, REGAIN recon was readily used to deblur low-resolution CS-reconstructed tagging images without modifying the pre-trained network. REGAIN was implemented inline (i.e., in-scanner). Thus, the 12-fold accelerated k-space tagging data were reconstructed inline in real-time, and images were available upon completion of the scan (Fig. 2b).

## Evaluation

### Population

**Fig. 3:**
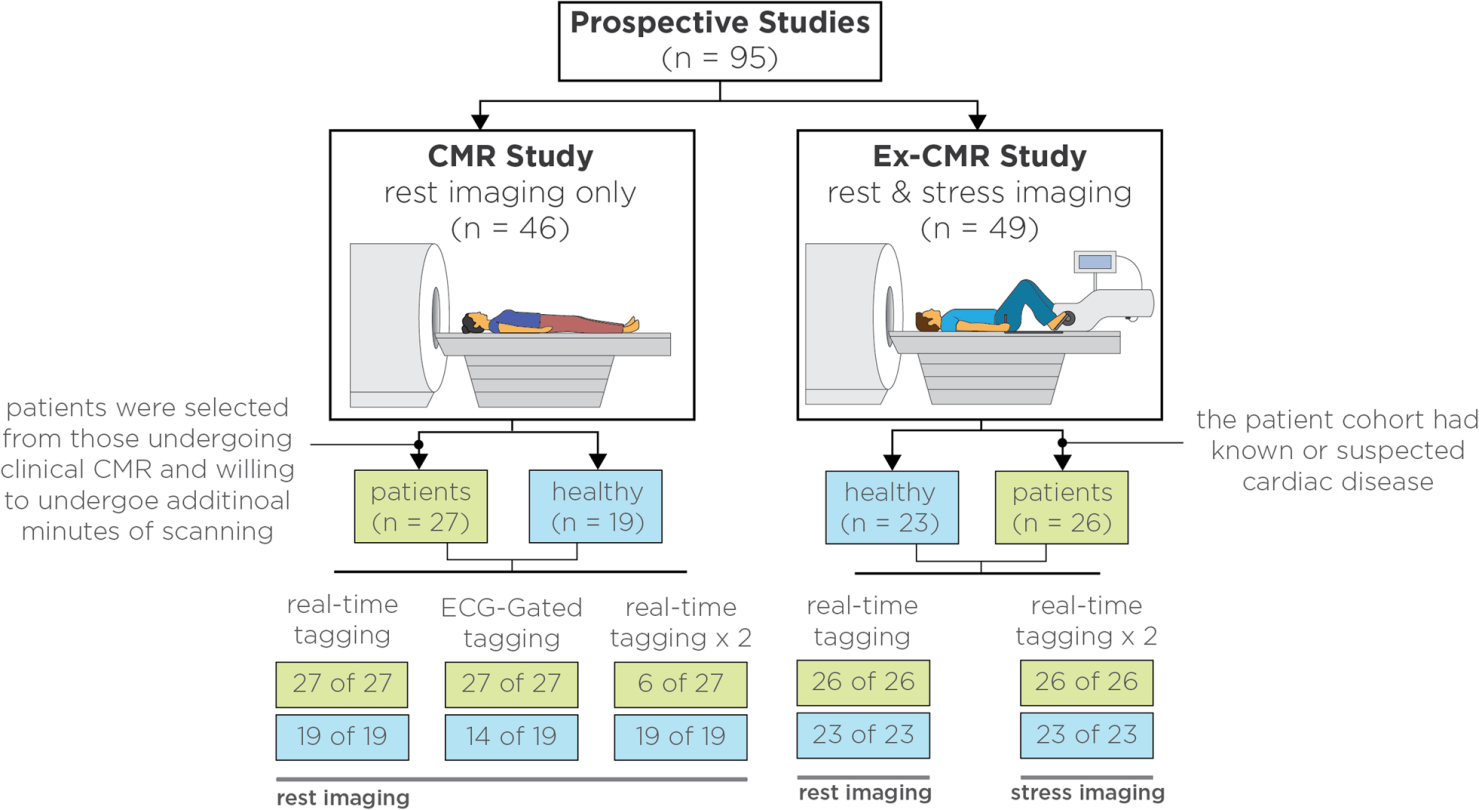
Prospective studies. A total of 95 subjects were prospectively recruited to evaluate our proposed technique. In the first study, CMR imaging was performed exclusively at rest in 27 patients and 19 healthy subjects. Patients were selected from those undergoing clinical CMR and willing to undergo an additional 5–10 min of scanning. Real-time tagging images were collected in all subjects. To serve as comparison, ECG-gated tagging images were also collected in all patients and a subset of healthy subjects (14 of 19). To assess repeatability, an extra set of real-time tagging images was collected in a subset of patients (6 of 27) and all healthy subjects. In the Ex-CMR study, real-time tagging images were collected in all subjects before and after supine bike exercise. *ECG* electrocardiogram, *Ex-CMR* exercise cardiac magnetic resonance

Two prospective studies were conducted to assess the proposed technique (Table 1). All subjects provided informed consent approved by the Institutional Review Board for using their medical information in research. All patient data were handled in accordance with the Health Insurance Portability and Accountability Act. In the first study (Fig. 3), we focused on evaluating the technique exclusively at rest by recruiting 27 patients (19 male, 54 ± 16 years) and 19 healthy subjects (7 male, 26 ± 4 years). Patients were selected from those undergoing clinical CMR and willing to undergo an additional 5–10 min of scanning. All patients were eligible except for those with cardiovascular implantable electronic devices. CMR technologists approached patients based on time and staffing availability for consent. In the second study, we assessed the feasibility of real-time tagging imaging under physiological stress by recruiting 26 patients (18 male, 55 ± 15 years) and 23 healthy subjects (10 male, 40 ± 14 years) for an exercise-CMR study. The patient cohort included individuals with various heart failure types and LV ejection fraction levels, as well as those with shortness of breath under evaluation. Patients with significant valvular disease or cardiovascular implants were excluded.

**Table 1:** Subject demographics

	CMR		Exercise CMR	
	Healthy	Patient	Healthy	Patient
Subjects	19	27	23	26
Sex				
Male	7 (37%)	19 (70%)	10 (43%)	18 (69%)
Female	12 (63%)	8 (30%)	13 (57%)	8 (31%)
Age (years)^a^				
Male	27 ± 4 (26–30)	56 ± 15 (48–68)	35 ± 12 (24–43)	57 ± 14 (52–63)
Female	25 ± 4 (21–28)	50 ± 20 (39–58)	43 ± 15 (32–58)	52 ± 16 (44–59)
Weight (kg)	69 ± 14	87 ± 18	68 ± 12	85 ± 15
Height (cm)	168 ± 11	177 ± 10	171 ± 11	174 ± 9
Body Mass Index (lbs/m^2^)	24 ± 3	28 ± 6	23 ± 4	28 ± 5

Continuous data are presented as mean ± standard deviation; categorical data are presented as number with percentages in parenthesis

^a^Data in parenthesis represent interquartile range

### Image acquisition

**Fig. 4:**
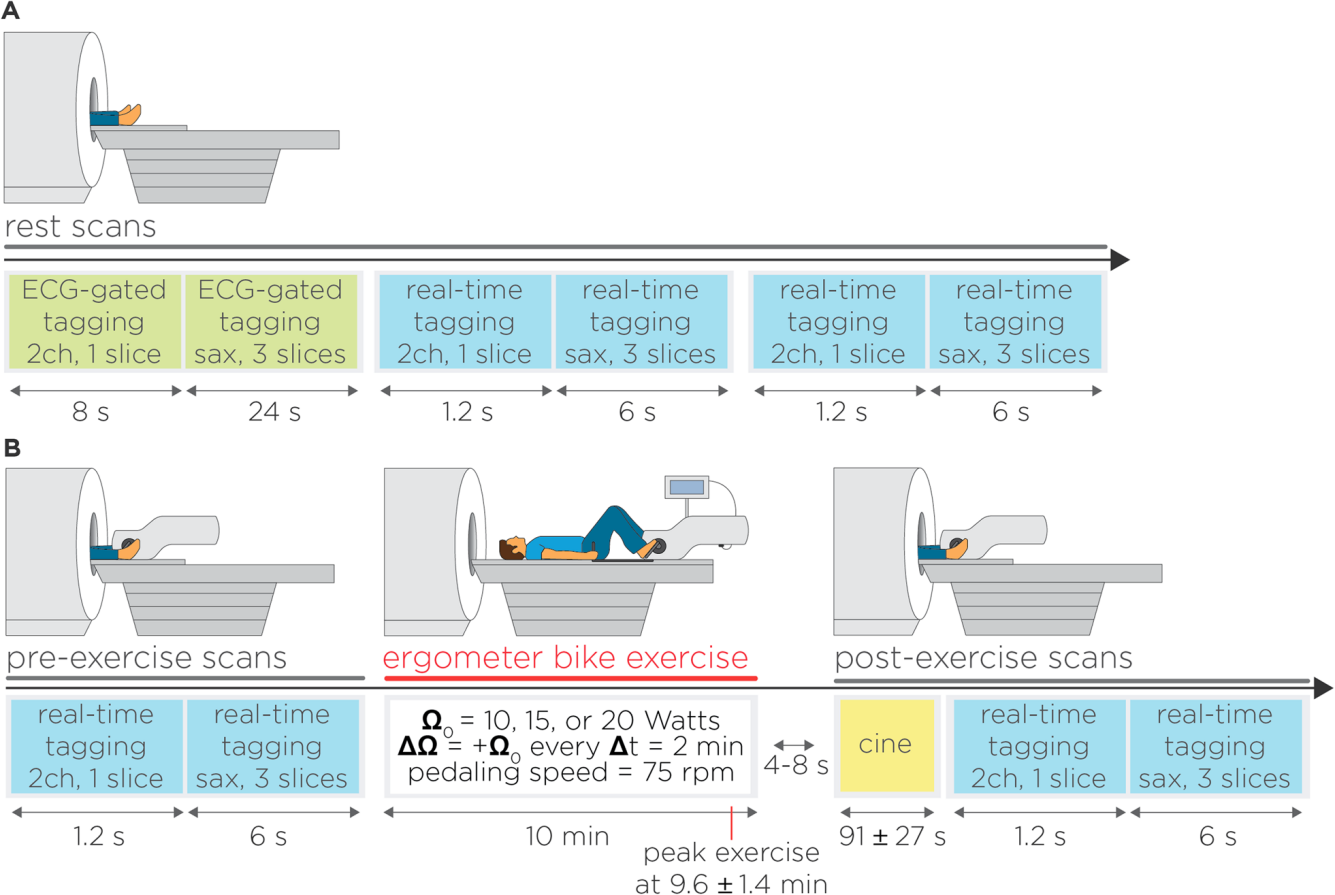
CMR and exercise CMR imaging protocols. **A** Our technique was first evaluated at rest, which consisted of breath-hold, electrocardiogram (ECG)-segmented tagging followed by free-breathing real-time tagging. The real-time acquisition was repeated once to assess repeatability. **B** Feasibility of real-time tagging imaging with physiology stress was evaluated in subjects prospectively recruited for exercise CMR. Real-time tagging images were collected pre- and post-exercise. Subjects were exercised in the supine position using a cycle ergometer. Work rate was started at Ω_0_ = 10W, 15W, 20W or 25W, and was increased by ∆Ω_0_ = + Ω_0_ every 2 min while subjects maintained a constant pedaling speed of 75 rpm. After 10 min or exhaustion, subjects were immediately transferred for post-exercise stress imaging. This consisted of a repetition of the real-time tagging sequence

Imaging was performed at 3 T using a commercial 18-channel cardiac coil and a 12-channel spine array (MAGNETOM Vida Siemens Healthineers, Erlangen, Germany). In the first study, we collected real-time tagging images in all subjects. To serve as comparison, ECG-segmented images were also collected in all patients (27 of 27) and a subset of healthy subjects (14 of 19). To assess repeatability, an extra set of real-time tagging images was collected in a subset of patients (6 of 27) and all healthy subjects (19 of 19) (Fig. 4A). In the Ex-CMR study, real-time tagging images were collected in all subjects before and after supine bike exercise (Fig. 4B). Subjects were exercised outside the scanner bore using a CMR-compatible cycle ergometer (Lode, Groningen, The Netherlands) attached to the scanner table. The work rate was started at Ω and increased by ∆Ω = + Ω every 2 min while subjects maintained a constant pedaling speed of 75 rpm. Subjects exercised with Ω = 10–25W resistance protocols. Heart rate during exercise was measured using a pulse oximeter at the end of each incremental step. Immediately after reaching the target heart rate ([220 − age] × 0.85), exhaustion, or after 10 min, subjects were returned to the scanner bore for post-exercise stress imaging. There was 4–8 s gap between the end of exercise and stress imaging. Further, cine images were collected prior to tagging to evaluate left and right ventricular function immediately post-exercise but they are not reported as part of this study. Therefore, this resulted in an additional delay of 91 ± 27 s prior to tagging imaging.

### Image analysis

In the first study, we aimed to investigate the feasibility of quantifying myocardial deformation using real-time tagging images. We employed an open-source DL model for landmark tracking to generate measures of mid-wall global circumferential strain (GCS) [44]. The DL model has been previously trained for short-axis tracking. Therefore, deformation was only quantified in short-axis images. Each slice was analyzed separately, with a global value obtained by averaging strain across slices. The strain was derived from ECG-segmented and real-time images at rest to assess agreement and from repeated real-time acquisitions to assess repeatability.

In the Ex-CMR study, we aimed to assess the feasibility of real-time tagging imaging during physiological stress using real-time tagging images collected at rest and after supine exercise. Two experienced CMR readers (F.G: 5 years, S.N: 14 years) independently evaluated the quality of 2-chamber and short-axis images. The quality of all real-time images was evaluated, including those collected at rest in the first study. The readers were blinded to the timing of acquisition and were presented with the images separately and in random order. Each reader independently rated image quality on a 4-point Likert scale based on tagline quality and artifact level. Tagline quality was graded as follows: 1-excellent, with well-defined tagline patterns and clear differentiation between dark and bright lines; 2-good, with well-defined patterns but with partial signal intensity in saturated lines; 3-moderate, with distinguishable but not well-defined patterns and partial signal intensity in saturated lines that appeared grey. Taglines also showed minor motion-induced blurring; 4-poor, with no well-defined patterns, significant brightness in saturated regions, and significant respiratory and cardiac motion-induced blurring. Artifact level was graded as follows: 1-none, with no artifacts present in the image; 2-minimal, with slight artifacts present that did not significantly impact image quality; 3-moderate, with noticeable artifacts present that somewhat degrade image quality; 4-significant, with severe artifacts rendering the image unusable for diagnostic purposes. Lastly, the feasibility of quantifying myocardial deformation after physiological exercise was assessed by evaluating mid-wall GCS in real-time tagging images post-exercise.

### Statistical evaluation

The SciPy (stats, v1.7.1) package was used for statistical analysis [46]. We assessed agreement between ECG-segmented and real-time measures of mid-wall GCS using linear regression, constraining the intercept to zero, and expressed it as slope [95% confidence interval (CI)]. The correlation was evaluated using the Pearson correlation coefficient, r, with the following criteria: r values above 0.8 indicated a very strong correlation, above 0.7 good, above 0.6 moderate, above 0.3 fair, and below 0.3 poor [47]. Scan/re-scan reproducibility of real-time tagging mid-wall GCS measures was assessed using the coefficient of variation (CoV) and intraclass correlation coefficient (ICC). Based on the ICC [95% CI] estimate, values less than 0.5, between 0.5 and 0.75, between 0.75 and 0.9, and greater than 0.90 were considered poor, moderate, good, and excellent reliability, respectively. The minimal detectable change was defined as standard measurement error × $\sqrt{2}\times 1.96$, where the standard measurement error is given by standard deviation × $\sqrt{1-\text{ICC}}$ and the standard deviation is based on the measurements from the first scan. Differences in GCS were also visualized using Bland–Altman plots and expressed as mean difference [mean difference ± 1.96 × standard deviation of the difference].

## Results

Real-time tagging images were successfully acquired and reconstructed in all subjects. At rest, the quality of 2-chamber images was diagnostic in 97% of subjects (92 of 95) and 90% of subjects (44 of 49) post-exercise. In the other 8 subjects, 2-chamber images were excluded from the analysis due to incorrect slice prescription. The image quality of all short-axis images was assessed. Among patients, 1 was found to have atrial fibrillation, and 2 exhibited isolated monomorphic premature ventricular contractions at the time of the scan. All healthy subjects and 96% of patients (25 of 26) completed the exercise protocol, achieving the maximal exercise and heart rate after 9.6 ± 1.4 min (Table 2). One patient terminated the protocol early because of leg fatigue.

**Table 2:** Response to exercise CMR protocol

	Healthy	Patient
Subjects (n)	23	26
Workout time (min)	9.6 ± 1.1	9.6 ± 1.6
Maximal exercise intensity (W)	76 ± 16	56 ± 20
Resting HR (beats/min)	68 ± 10	67 ± 13
Resting SBP (mmHg)	112 ± 14	115 ± 14
Maximal HR during exercise (beats/min)	123 ± 14	104 ± 15
Maximal SBP during exercise (mmHg)	145 ± 18	149 ± 21
Maximal ΔHR during exercise (beats/min)	55 ± 16	37 ± 15
Maximal ΔSBP during exercise (mmHg)	33 ± 15	34 ± 16
Recovery heart rate (beats/min)	73 ± 12	72 ± 17
Recovery systolic (mmHg)	108 ± 10	113 ± 14
HR during tagging imaging (beats/min)	94 ± 18	81 ± 15

Continuous data are presented as mean ± standard deviation

*CMR* cardiac magnetic resonance; *HR* heart rate, *SBP* systolic blood pressure

**Fig. 5:**
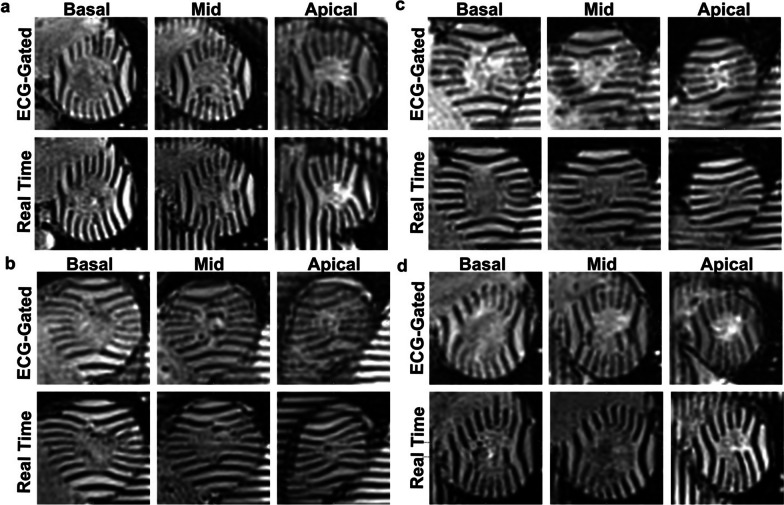
Breath-hold ECG-segmented and free-breathing real-time tagging imaging. Steady-state free-precession tagging images were collected in patients (**a**–**c**) and a healthy subject (**d**) at 3 T. Tag lines with 8 mm thickness were oriented 90° perpendicular to the phase encoding, which resulted in horizontal and vertical lines. Three slices at basal, mid, and apical levels were acquired. Imaging parameters for both ECG-segmented and real-time acquisitions were: matrix size = 176 × 176, acquired spatial resolution = 4.0 × 2.0 mm^2^, reconstructed spatial resolution = 2.0 × 2.0 mm^2^_,_ slice thickness = 8 mm, slice distance factor = 150%, echo and repetition time = 1.1/2.62 ms, temporal resolution = 28.8 ms and flip angle = 30°. Images are shown at end-systole. *ECG* electrocardiogram

**Fig. 6:**
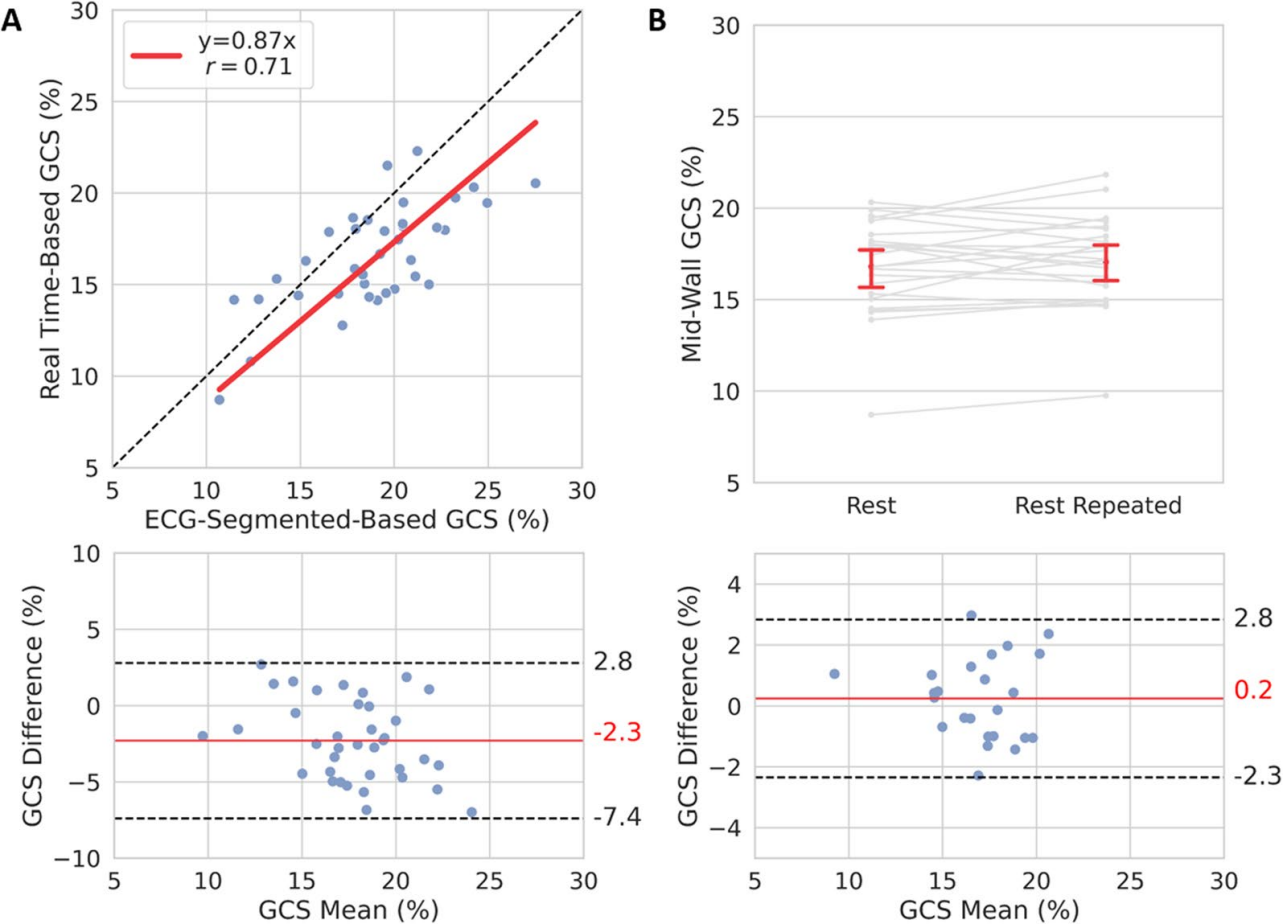
Evaluation of mid-wall global circumferential strain (GCS). Tagging images were collected at rest with breath-hold ECG-segmented and with repeated free-breathing real-time tagging sequences. Measurements of mid-wall GCS were obtained from tagging images in the short-axis view at basal, mid and apical levels and were averaged across slices. **A** The average strain obtained with the two different sequences was compared. Linear regression with forced zero intercept had a slope = 0.87 (0.83, 0.91), and Pearson correlation coefficient r was 0.71. Bland–Altman analysis showed a mean difference of − 2.3% (− 7.4, 2.8). **B** The average strain obtained from repeated real-time tagging sequence was compared to evaluate intra-subject repeatability of mid-wall GCS measures. Intraclass correlation coefficient was 0.86 (0.71, 0.94). The Bland–Altman analysis showed a mean difference of 0.2% (− 2.3, 2.8). In Bland–Altman plots, limits of agreement are shown as dotted black lines, and biases as solid red lines

In the first study, where CMR was performed exclusively at rest, myocardial deformation was successfully quantified from ECG-segmented and real-time images in 90% of cases (37 of 41) (Fig. 5). Data excluded from analysis were due to poor ECG-segmented (2 of 4) or real-time (1 of 4) image quality or poor tracking in real-time images (1 of 4). A good correlation (*r* = 0.71) was observed between ECG-segmented and real-time measurements of mid-wall GCS. Linear regression showed an 87% [0.83, 0.91] agreement between sequences. Bland–Altman analysis showed a mean difference of − 2.3% (− 7.4, 2.8) (Fig. 6a). GCS measures from the repeated real-time images had a CoV = 4.7% and good to excellent repeatability (ICC = 0.86 [0.71, 0.94]). The minimal detectable change in GCS was 2.6%. Bland–Altman analysis showed a mean difference of 0.2% (− 2.3, 2.8) (Fig. 6b).

**Fig. 7:**
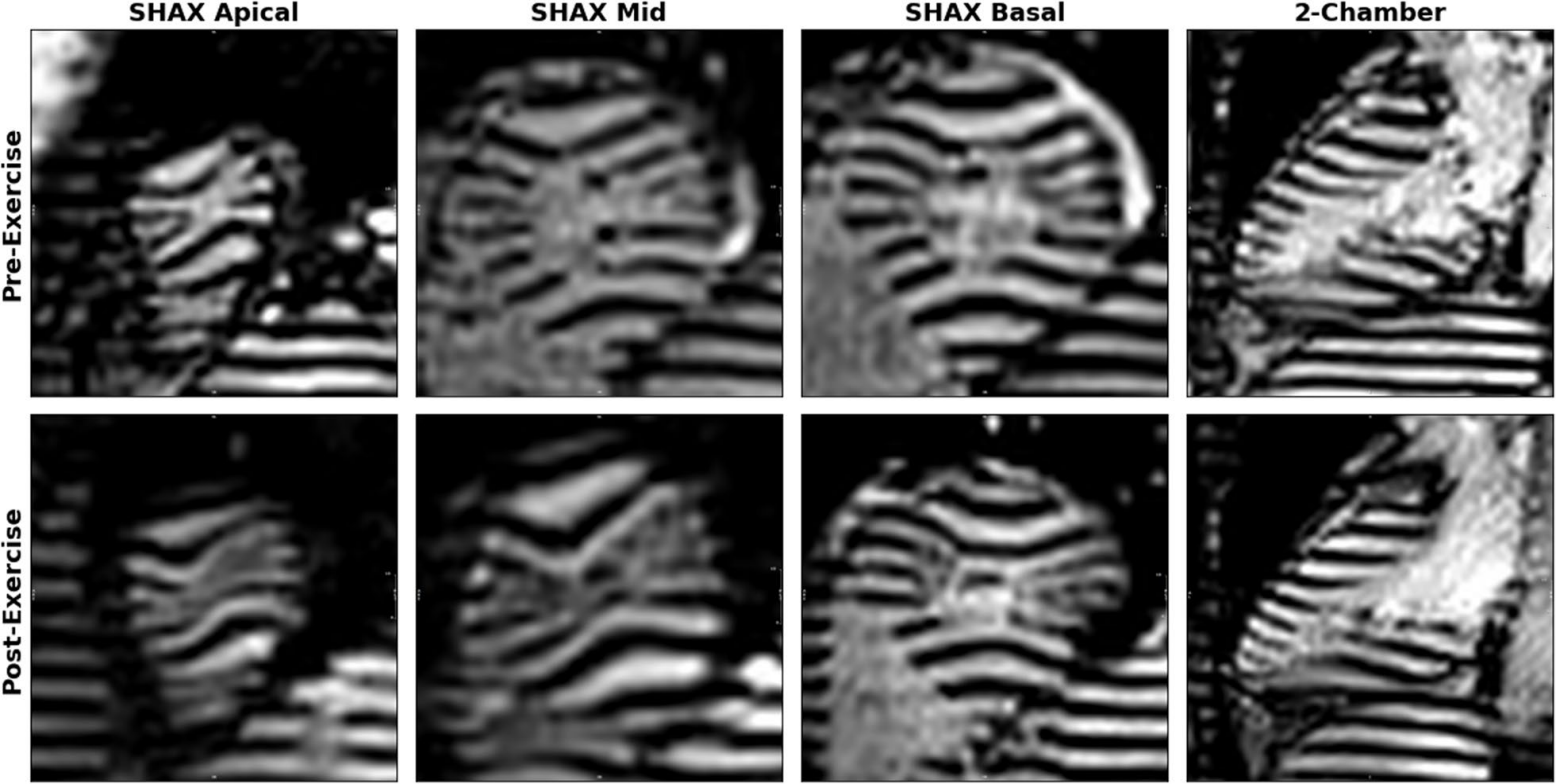
Clinical case illustrating microvascular dysfunction. A 54-year-old female presenting with persistent chest pain without angina symptoms or ischemic ECG changes on the stress test. Coronary angiography revealed a right dominant system with catheter-induced vasospasm in the right coronary artery and evidence of microvascular dysfunction. Following a 10-min exercise CMR protocol with a 15-W resistance increase every 2 min, her heart rate increased from 56 to 130 bpm. Real-time tagging CMR images were acquired pre- and post-exercise in short-axis and 2-chamber views. Tagging line quality for all acquisitions in both views was excellent (score = 1), characterized by well-defined tagline patterns with clear differentiation between dark and bright lines. Post-exercise tag lines were markedly deformed. All images shown near end-systole. *ECG* electrocardiogram

**Fig. 8:**
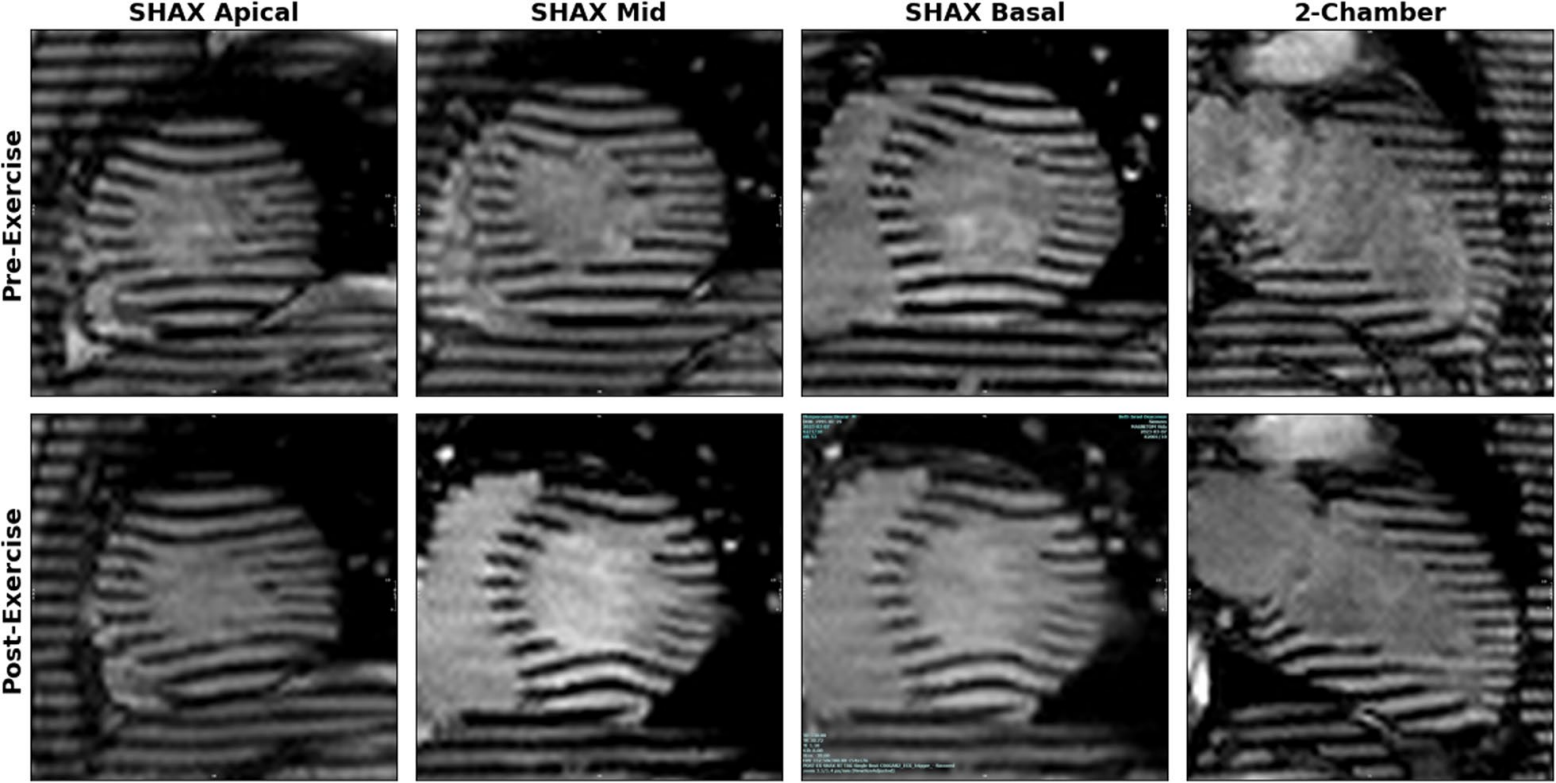
Clinical case of heart failure. A 29-year-old male with a history of heart failure and an ejection fraction of 33%. After a 10-min exercise CMR protocol with a 10-W resistance increase every 2 min, his heart rate increased from 80 to 116 bpm. Real-time tagging images were acquired pre- and post-exercise in short-axis and 2-chamber views. Tagging line quality for all acquisitions in both views was good (score = 2), defined as a well-defined pattern but with partial signal intensity in saturated lines. Tagging lines demonstrated a moderate increase in myocardial deformation post-exercise. *CMR* cardiac magnetic resonance

**Fig. 9:**
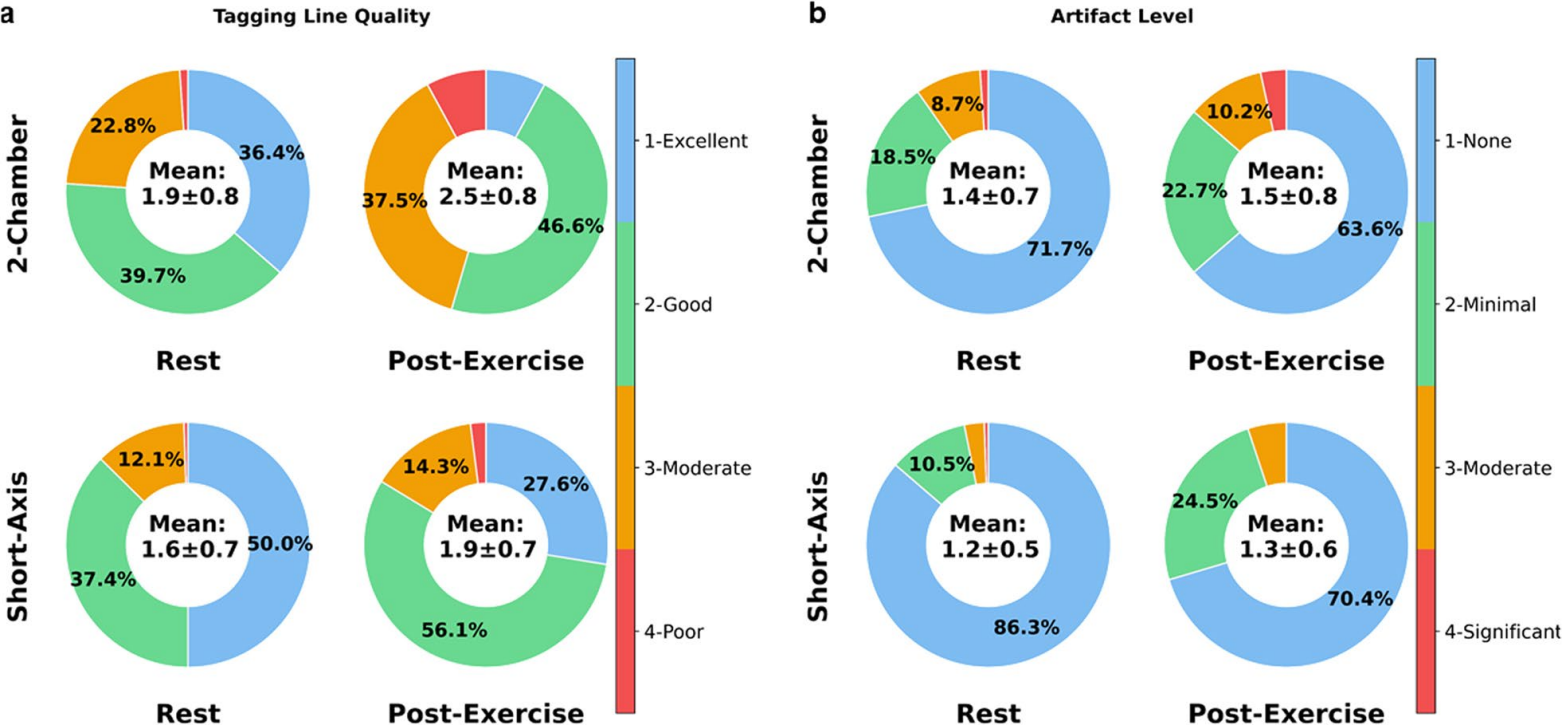
Subjective evaluation of image quality. Short-axis images from 95 subjects at rest and 49 subjects post-exercise were evaluated. For 2-chamber images, scans from a total of 92 subjects at rest and 44 subjects pre-exercise were assessed. Therefore, there were a combined total of 280 scans, including 2-chamber and short-axis images. Two readers separately rated data on a 4-point Likert scale based on tagline quality (1-excellent; 2-good; 3-moderate; 4-poor) and artifact level (1-none; 2-minimal; 3-moderate; 4-significant). **a** The tagging line quality score for 2-chamber and short-axis images at rest was 1.9 ± 0.8 and 1.6 ± 0.7. Post-exercise, the 2-chamber score increased to 2.5 ± 0.8, and the score for short-axis images increased to 1.9 ± 0.7. The artifact level for both 2-chamber and short-axis images pre-exercise was 1.4 ± 0.7 and 1.2 ± 0.5. Artifact level rose to 1.5 ± 0.8 and 1.3 ± 0.6 for 2-chamber and short-axis images, respectively

Of the 280 scans, each subjectively evaluated by two readers, 35% of scans (196 of 560) were graded as having excellent tag quality (i.e., score = 1). A clinical case with excellent rest and post-exercise image quality is presented in Fig. 7 and Additional file 1: Video S1. In 43% of scans (240 of 560), tag quality was graded as good (i.e., score = 2), represented at rest and post-exercise in Fig. 8 and Additional file 2: Video S2. Specifically, the quality score for 2-chamber and short-axis images collected at rest was 1.9 ± 0.8 and 1.6 ± 0.7, with less than 3% of scores graded poor (Fig. 9). Post-exercise, the score of 2-chamber images was to 2.5 ± 0.8, and the number of scores graded as poor was 8.0%. Tag quality of short-axis images after exercise was 1.9 ± 0.7, with scores graded as poor being less than 3%. The artifact level was mainly influenced by off-resonance and banding artifacts. For both 2-chamber and short-axis images taken at rest, artifact level was 1.4 ± 0.7 and 1.2 ± 0.5, with less than 3% graded as poor. Artifact level score post-exercise was 1.5 ± 0.8 and 1.3 ± 0.6 for 2-chamber and short-axis images, respectively. The number of scores graded poorly after exercise was less than 3% in both views.

**Fig. 10:**
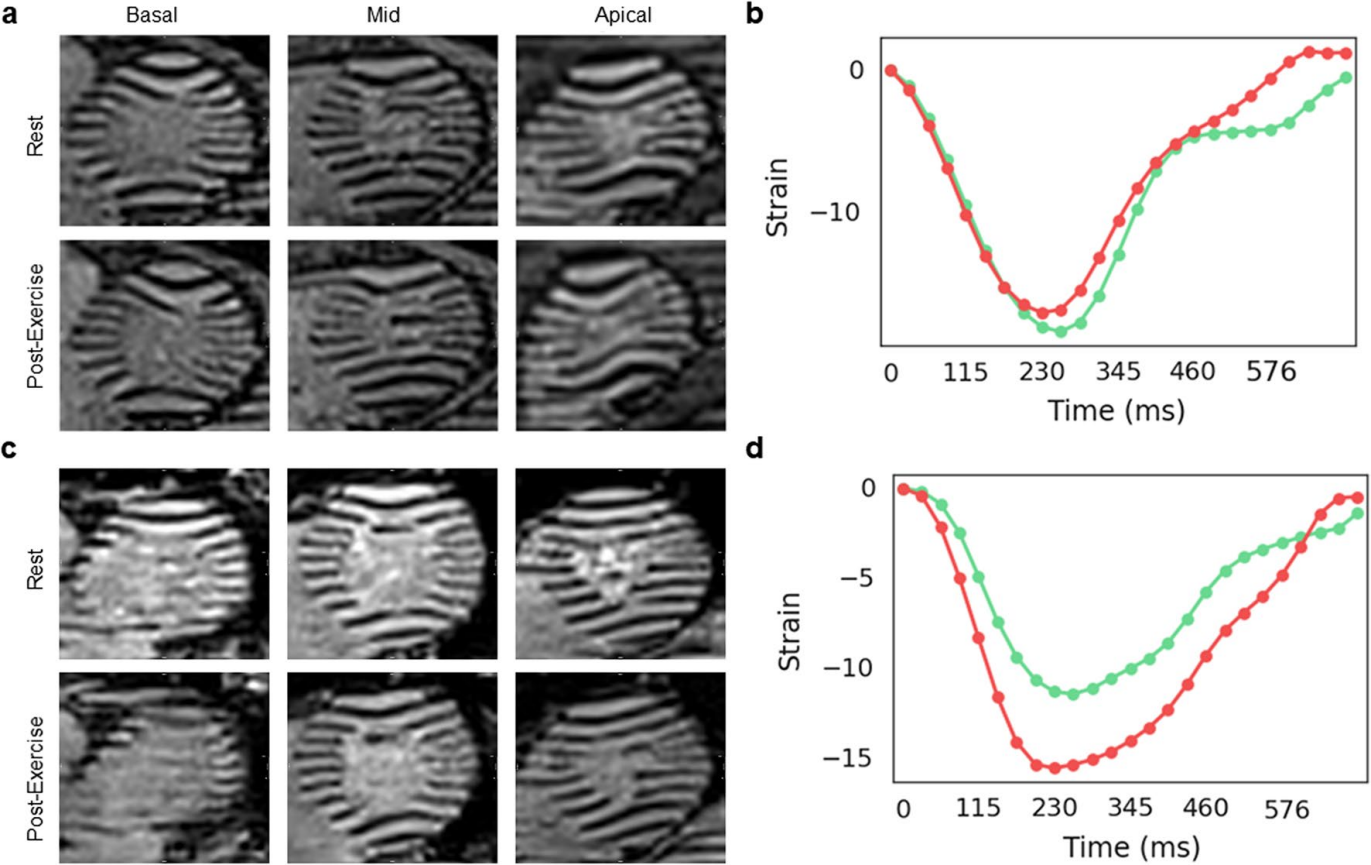
Quantification of myocardial deformation in Ex-CMR real-time tagging imaging. **a** A 55-year-old male with a history of heart failure with preserved ejection fraction. Real-time tagging images were acquired pre- and post-exercise in short-axis views. Quantification of myocardial deformation shows a minimal response. **b** A 63-year-old male with a history of heart failure with reduced ejection fraction. Quantification of showed an augmented myocardial deformation

Quantifying myocardial deformation from real-time tagging images was achieved in 96% of subjects (47 of 49) pre-exercise and 84% of subjects (41 of 49) post-exercise. Quantification was not feasible due to prominent respiratory motion in five cases and poor image quality in two cases. In three cases post-exercise, the first acquired cardiac phase was near end-systole, which prevented proper quantification of strain. Mid-wall GCS for two patients with heart failure is shown in Fig. 10.

## Discussion

In this study, we presented a real-time myocardial tagging imaging technique based on bSSFP readout with inline low-latency DL reconstruction. Our results demonstrate the feasibility of real-time tagging imaging for quantifying myocardial deformation at rest and during physiological stress, showing good agreement and repeatability between ECG-segmented and real-time measurements of mid-wall GCS at rest. Most cases pre- and post-exercise had a good to excellent tagline quality with minimal to no artifacts.

Standard line and grid tagging with gradient-echo readout are available in vendor platforms. However, current gradient-echo sequences are not robust for imaging during exercise stress. Previous pharmacological [12–14] and exercise [25, 26] studies used ECG-segmented sequences that required breath-holding. In those studies, the sequences were implemented at 1.5 T [11–14, 25, 26]. In the present study, we proposed a free-breathing, highly accelerated real-time tagging sequence. Although tagline quality was slightly lower post-exercise, most notably in the 2-chamber view, most images remained usable for myocardial deformation quantification. Further optimization may be necessary, and clinical evaluation studies in various patient populations are warranted. In addition, both 2-chamber and short-axis slices could instead be acquired during a single breath-hold, which could be advantageous to improve consistency in quantification and improve tagline quality.

The REGAIN-based twofold and CS-based sixfold image acceleration enabled us to achieve a nominal 12-fold acceleration rate with 2.0 × 2.0 mm^2^ spatial resolution after reconstruction and 28.8 ms temporal resolution without sliding windows. This is a significant advance compared to previous studies that have reported acceleration rates 3–8 [33, 45, 48]. The REGAIN-based undersampling method is based on limiting the maximum k_y_ lines. This approach removes the high-frequency components along the PE direction containing spatial harmonics in grid tagging. Therefore, we only placed line tags orthogonal to the readout direction. In addition, acceleration based on reducing the spatial resolution limits the minimum tagging line spacing, which in this study was 8 mm. Nevertheless, the REGAIN model was based entirely on cine data. Therefore, the model could be re-trained with tagging data to address these challenges. Moreover, alternative sequence designs could explore extending our current technique to a three-dimensional imaging [49].

We implemented the proposed sequence using bSSFP imaging to achieve increased contrast, tagging persistence, and to trade the SNR gain for higher acceleration and improved temporal resolution [40–42, 50]. One challenge of bSSFP-based tagging is its sensitivity to off-resonance effects. These effects are worse at 3 T. We mitigated these effects using linearly increasing ramp-up pulses. The number of ramp-up pulses could be increased to minimize artifacts further. However, for post-exercise imaging, increased ramp-up pulses would compromise the acquisition of the early cardiac cycle. Nevertheless, we found that five pulses were sufficient to suppress artifacts, as previously reported by Herzka et al. [42] and demonstrated here by the minimal to no artifacts scoring. However, the shimming volume pre-exercise should be carefully adjusted and large enough to prevent banding artifacts post-exercise.

In this study, we encountered two primary challenges that could impact the quantification of myocardial deformation from real-time tagging images. First, because we placed the tagging imaging block immediately after detecting a single R-wave cardiac trigger, image acquisition starts after the heart has already begun to contract. Such imaging delay could hinder accurate quantification at high heart rates. To address this challenge, future investigations could use adaptive triggering to initiate ramp-up pulses and the tagging preparation module before the cardiac trigger, ensuring a more precise evaluation of deformation under post-exercise conditions. In addition, blood suppression could enable proper delineation of the myocardial wall boundaries. The second challenge was quantification based on the pre-trained CNN proposed by Loecher et al. [44]. Although this quantification approach resulted in good to excellent repeatability with a relatively small minimal detectable change, this tracking method was exclusively trained using short-axis images, which constrained our analysis to this view and precluded the examination of 2-chamber images. Furthermore, the CNN was trained with simulated CMR data using gradient-echo parameters and featuring elliptical cardiac contraction. However, cardiac motion post-exercise stress exhibits increased complexity as well as substantial respiratory motion. Additionally, our sequence was based on bSSFP. Further studies may be needed using a CNN model trained with bSSFP parameters and featuring more complex cardiac contraction patterns and free-breathing respiratory motion. Real-time tagging images collected in this study as well as the REGAIN DL reconstruction model are made available online.

In this study, we also showed examples that underline the potential of quantifying myocardial deformation after exercise. Such evaluation holds clinical value for characterizing the response of heart failure patients to exercise. A diminished increase in mid-wall GCS in patients suffering from heart failure may suggest compromised contractility, thereby contributing to a decreased stroke volume reserve. However, since strain is recognized as being load-dependent, these findings warrant further comprehensive analysis in a more extensive population of heart failure patients. This will help ascertain the value of strain as a supplementary measure to volumetric and functional evaluations under physiological stress.

## Limitations

The present report represents a feasibility study with a small sample size. Since subjects were exercised outside of the scanner bore, imaging was not performed during peak exercise. Stress perfusion was not included as part of the imaging protocol for this study. The proposed tagging technique was evaluated using lines only, and in its current form, the model does not support grid tagging. Quantification of beat-to-beat variation was performed in the short axis only. Only three slices were acquired. Therefore, mid-wall GCS does not represent the whole volume. We did not evaluate the inter- or intra-observer reproducibility of measures of mid-wall GCS. Finally, data from a single vendor and field strength were used for REGAIN training, and the generalizability of the proposed tagging technique should be studied.

## Conclusion

We developed and demonstrated the feasibility of a highly accelerated bSSFP real-time tagging with 28.8 ms temporal resolution for Ex-CMR at 3 T.

### Supplementary Information

**Additional file 1: Video S1.** Clinical case illustrating microvascular dysfunction. Following an exercise CMR protocol with a 15-W resistance increase every 2 min, the heart rate increased from 56 to 130 bpm. Real-time tagging CMR images were acquired pre- and post-exercise in short-axis and 2-chamber views. Tagging line quality for all acquisitions in both views was excellent (score = 1), characterized by well-defined tagline patterns with clear differentiation between dark and bright lines.

**Additional file 2: Video S2.** Clinical case of heart failure. Following an exercise CMR protocol with a 10-W resistance increase every 2 min, heart rate increased from 80 to 116 bpm. Real-time tagging images were acquired pre- and post-exercise in short-axis and 2-chamber views. Tagging line quality for all acquisitions in both views was good (score = 2), defined as a well-defined pattern but with partial signal intensity in saturated lines.

## Data Availability

The proposed reconstruction model is an investigational technique and not available by the vendor as a research tool or product. The model codes are openly available on GitHub: https://github.com/HMS-CardiacMR/REGAIN. The proposed tagging sequence is an investigational technique and not available by the vendor as a research tool or product. Real-time tagging datasets collected in this study as well all quantitative and qualitative analysis supporting the conclusions of this article are available in the Harvard Dataverse: https://dataverse.harvard.edu/dataverse/cardiacmr; 10.7910/DVN/JMZHVI.
