# Supplementary figures and images for: Highly accelerated free-breathing real-time myocardial tagging for exercise cardiovascular magnetic resonance
Source: J Cardiovasc Magn Reson. 2023 Oct 2;25:56. doi: 10.1186/s12968-023-00961-w (PMC10544487; doi:10.1186/s12968-023-00961-w)

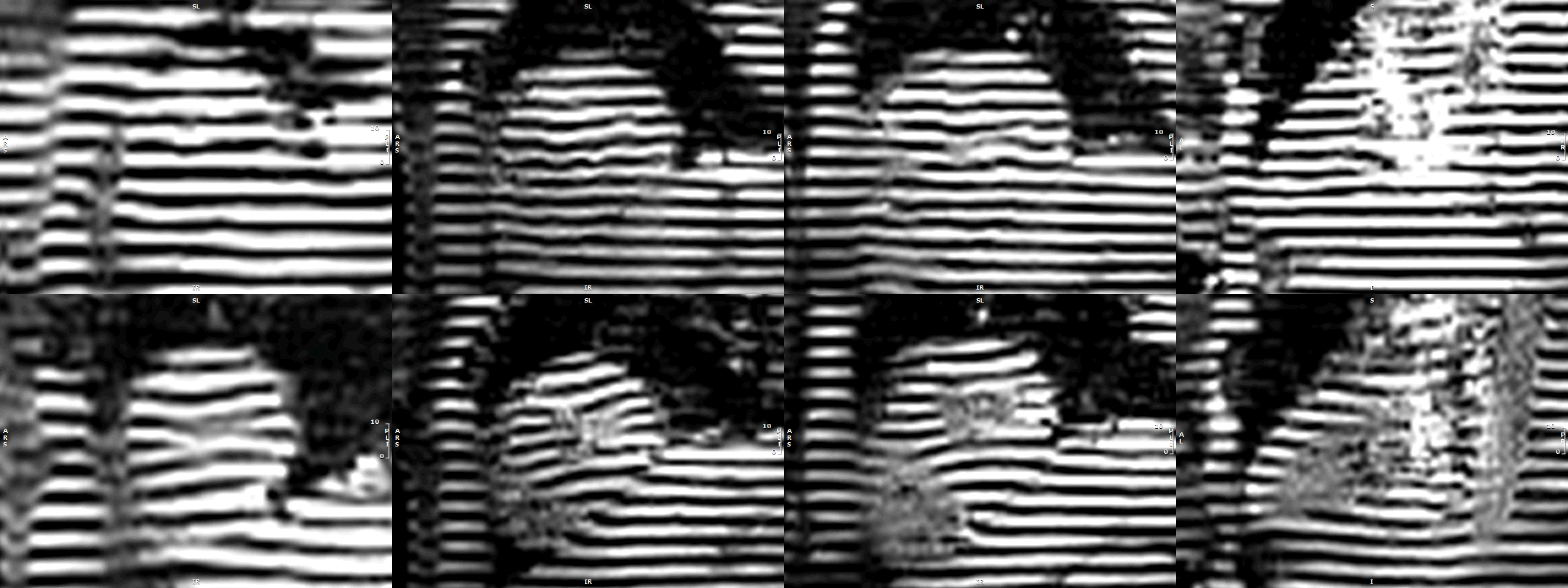

Supplement: Supplementary file 1 — Additional file 1: Video S1. Clinical case illustrating microvascular dysfunction. Following an exercise CMR protocol with a 15-W resistance increase every 2 min, the heart rate increased from 56 to 130 bpm. Real-time tagging CMR images were acquired pre- and post-exercise in short-axis and 2-chamber views. Tagging line quality for all acquisitions in both views was excellent (score = 1), characterized by well-defined tagline patterns with clear differentiation between dark and bright lines. [file 12968_2023_961_MOESM1_ESM.gif]

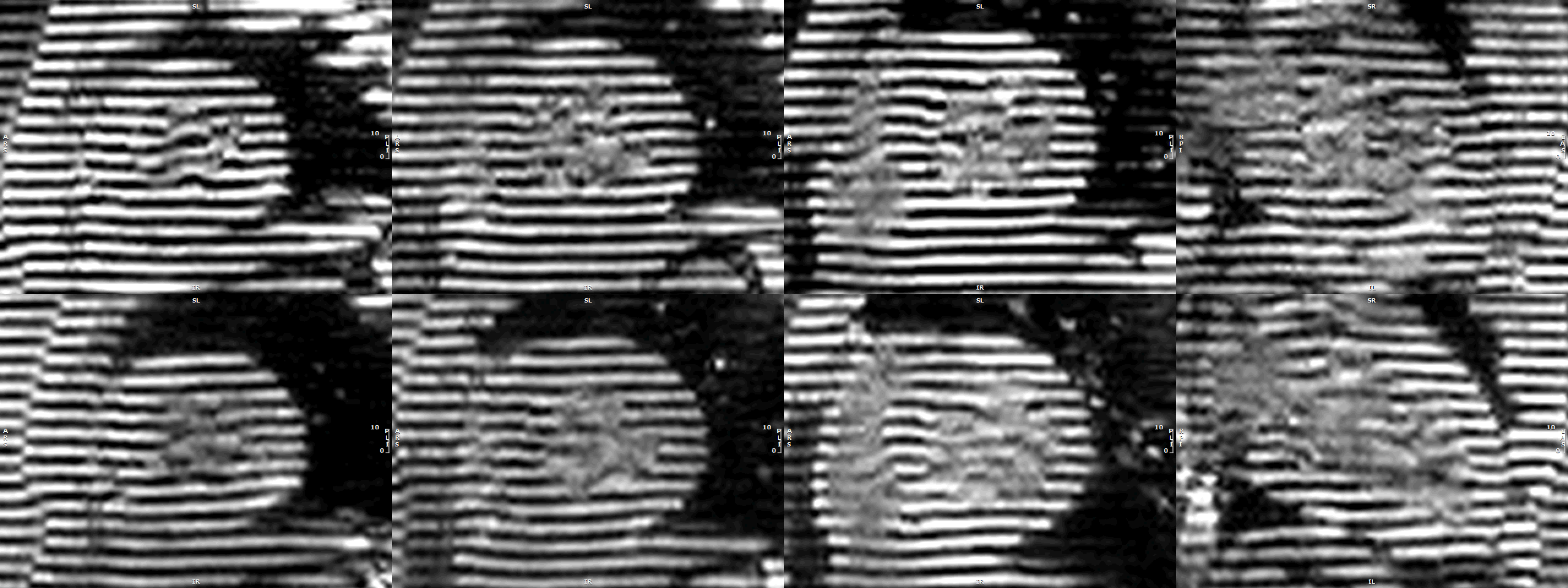

Supplement: Supplementary file 2 — Additional file 2: Video S2. Clinical case of heart failure. Following an exercise CMR protocol with a 10-W resistance increase every 2 min, heart rate increased from 80 to 116 bpm. Real-time tagging images were acquired pre- and post-exercise in short-axis and 2-chamber views. Tagging line quality for all acquisitions in both views was good (score = 2), defined as a well-defined pattern but with partial signal intensity in saturated lines. [file 12968_2023_961_MOESM2_ESM.gif]
